# Risk Factors in Outpatients with Dermatitis and Eczema in Tertiary Hospitals of China Who Have Clinically Suspected Bacterial Infection

**DOI:** 10.1155/2020/7621217

**Published:** 2020-11-27

**Authors:** Yan Li, Wei Xu, Linfeng Li

**Affiliations:** Department of Dermatology, Beijing Friendship Hospital, Capital Medical University, Beijing 100050, China

## Abstract

Skin bacterial infections are often observed in eczema patients, but the risk factors are not fully understood. The current study evaluated the prevalence of clinically suspected bacterial infection and its associated risk factors. Moreover, we investigated the practice of skin infection diagnosis in China. A hospital-based, multicenter, cross-sectional epidemiologic survey of bacterial infection was performed in a total of 6208 outpatients diagnosed with dermatitis and eczema from 39 tertiary hospitals of 15 provinces and municipalities in China. All patients completed a specific questionnaire regarding their demographic characteristics, disease duration, distribution of lesions, severity of itching, and medical history. Univariate analysis and multivariate analysis were used to evaluate risk factors associated with bacterial infection in patients with different types of eczema. The prevalence of clinically suspected bacterial infection was 47.0% in patients with eczema. Compared to atopic dermatitis, widespread eczema (OR = 1.50, *P* < 0.001) and other eczema (OR = 1.42, *P* < 0.001) were more likely to suffer bacterial infection. The itching grade was positively associated with the infection (OR = 2.11, 7.04, and 12.3 in patients with mild, moderate, and severe itching, respectively; *P* < 0.001). Lesion distribution in the cubital fossa, popliteal fossa, ear, shoulder and back, axillary, foot, and pudendum was positively associated with bacterial infection (all OR > 1.0, *P* < 0.05). In contrast, the face and abdomen were reversely associated with bacterial infection (OR < 1.0, *P* < 0.005). History of asthma, allergic rhinitis, allergic conjunctivitis, atopic dermatitis, infantile eczema, and flexion dermatitis was positively associated with bacterial infection (all OR > 1.0, *P* < 0.005), while the history of dry skin was reversely associated with bacterial infection (OR = 0.76, 95% CI: 0.64-0.90; *P* = 0.002). Patients with eczema were easily infected with bacteria. Widespread eczema and other eczema were more likely to have bacterial infection than atopic dermatitis. The high rate of infection may attribute to the lack of corresponding bacterial detection, suggesting the need of guideline development in China to prevent overuse of topical antibiotics.

## 1. Introduction

Eczema is a common dermatologic disease that significantly affects patients' quality of life. Patients experience different degrees of itching and may have sleep disturbance [1]. Atopic dermatitis (AD) is a type of eczema; some people with AD have a personal and family history of allergies. It may be accompanied by asthma, allergic rhinitis, or other allergy-related disorders [2]. Skin bacterial infections are frequently observed in these patients, and invasive infections may occur if left untreated [3]. The most common skin infections in AD are caused by *Staphylococcus aureus* (*S*. *aureus*) and *Streptococcus pyogenes* (*S*. *pyogenes*) [4]. The risk factors for bacterial infections in eczema are not fully understood. It has been suggested that adult AD is particularly prone to secondary bacterial infection [5], possibly due to multiple scratches or erosion on the skin of these patients that leads to direct damage of the stratum corneum (SC) barrier, so that *S. aureus* can be easily feed with skin exudates after penetrating the skin [6]. Studies also suggested that loss-of-function mutations in the *filaggrin* (*FLG*) gene are a major cause for SC barrier disruption in patients with AD [7]. Malfunction of filaggrin may lead to penetration of foreign antigens and subsequent sensitization. However, another study of American patients with AD also did not find a significant association between *S*. *aureus* colonization and common *FLG* mutations [8]. Further study is required to clarify these conflicting findings and identify other risk factors for bacterial infections in eczema.

The abuse of antibiotics is a global problem, especially in China. Relevant policies and regulations have been developed in China to limit the use of systemic antibiotics, and improvement has been seen [9]. However, the abuse of topical antibiotics has not received much attention; we thus investigated the practice of the diagnosis of skin bacterial infections in China.

In this study, we performed a hospital-based, multicenter, cross-sectional epidemiologic survey of bacterial infection in outpatients with eczema. We found that clinically suspected bacterial infection was very common in patients with eczema. We also found that widespread eczema and other eczema were more likely to have bacterial infection than atopic dermatitis. Other risk factors for clinically suspected secondary bacterial infection in eczema included age of onset, course of disease, itching grade, lesion distribution, and history of dermatitis or eczema.

## 2. Methods

### 2.1. Study Population

A total of 6208 outpatients diagnosed with dermatitis and eczema were enrolled from 39 tertiary hospitals of 15 provinces and municipalities throughout China, including Anhui, Beijing, Chongqing, Guangdong, Henan, Hubei, Hunan, Liaoning, Jiangsu, Jiangxi, Shandong, Shanghai, Shanxi, Tianjin, and Zhejiang. This cross-sectional survey was carried out from March 1, 2014 to May 31, 2014. The patients were divided into two groups based on the presence or absence of clinically suspected bacterial infection. A total of 2918 patients with clinically suspected bacterial infection were in the bacterial group, and 3290 patients without bacterial infection were in the nonbacterial group. The demographic and clinical characteristics of the patients were summarized in Table 1. This study followed the rules of the Declaration of Helsinki and was approved by the Institutional Review Board Committee at each hospital. Written informed consent was obtained from each adult patient and the parents or guardians of each child patient, who knew the nature and possible consequences of the study.

### 2.2. Data Collection and Diagnosis Criteria

All dermatologists involved in this study received a standardized training for the project. Dermatological physical examination for each patient was independently performed by a dermatologist. All patients completed a specific questionnaire regarding their demographic characteristics, disease duration, distribution of lesions, severity of itching, and medical history. Itch was evaluated and divided into four levels: no itching, mild itching that did not interrupt daily life, moderate itching that interrupted daily activities but not affected sleep, and severe itching that affected both daily activities and sleep. History of allergic disease, dry skin, infantile eczema, and flexion dermatitis was also recorded. Allergic diseases included asthma, allergic rhinitis, allergic conjunctivitis, and atopic dermatitis.

In this study, atopic dermatitis was diagnosed according to the Williams diagnostic criteria [10]. Widespread eczema refers to the presence of eczema at multiple sites at the same time [11, 12].Other types of eczema, including allergic contact dermatitis, asteatotic eczema, autosensitization eczema, dyshidrotic eczema, hand eczema, irritant contact dermatitis, neurodermatitis, nummular eczema, photocontact dermatitis, seborrheic dermatitis, stasis dermatitis, unclassified eczema, and widespread eczema, were diagnosed on the basis of clinical characteristics and medical history according to the International Classification of Diseases- (ICD-) 10 [13–15]. Suspected secondary bacterial infection of dermatitis was clinically evaluated. The very likely bacterial infection was considered if superficial pustules, prudent exudation, or yellow-colored crust was detected. Suspected bacterial infection was considered if multiple scratches, oozing, erosion, or ulceration was found. In this study, the combination of the very likely bacterial and suspected bacterial infection was called bacterial infection. A previous study from our institute has demonstrated that the clinical diagnosis was highly correlated with the laboratory bacterial culture results; *S*. *aureus* was isolated in 92.9% of eczema patients who were diagnosed with very likely bacterial infection [16]. No laboratory test was performed to make the diagnosis [17, 18].

### 2.3. Statistical Analysis

All statistical analysis was performed using the SPSS software (version 22.0; IBM Corp., Armonk, NY). In univariate analysis, Student's *t*-test was used to compare the differences in age, age of onset, and course of disease between patients with bacterial infection and patients with no bacterial infection. Chi-square test was used to compare the differences in gender, diagnosis, itching grade, lesion distribution, and medical history between the two groups. In multivariate analysis, stepwise logistic regression was used to further evaluate associated risk factors for the suspected bacterial infection in patients with dermatitis and eczema. A significance level of 0.1 was required to allow a variable into the model or stay in the model. *P* < 0.05 was considered statistically significant.

## 3. Results

### 3.1. Demographic Characteristics of the Study Population and Prevalence of Clinically Suspected Bacterial Infection

The prevalence of patients with clinically suspected bacterial infection was 47.0% in all patients with dermatitis and eczema. No gender differences were found between the bacterial group and nonbacterial group (*P* = 0.14). Patients in the bacterial group were older in age (average age of 38.7 in the bacterial group and 36.9 years old in the nonbacterial group, *P* = 0.001).

### 3.2. Univariate Analysis of Clinically Suspected Bacterial Infection Associated with Risk Factors in Patients with Dermatitis and Eczema

The average onset age was significantly different between the two groups (35.6 vs. 34.1 years, *P* = 0.003; Table 1). The course of disease was significantly longer in patients with bacterial infection than those without bacterial infection (3.2 vs. 2.9 years, *P* < 0.0001). Compared with patients without bacterial infection, a significantly higher frequency of atopic dermatitis (17.4% vs. 14.2%, *P* = 0.0007) and widespread eczema (10.0% vs. 6.2%, *P* < 0.0001) was observed in patients with bacterial infection. In contrast, patients with bacterial infection had a significantly lower frequency of other eczema than those without bacterial infection (72.6% vs. 79.6%, *P* < 0.0001). Higher severity of itching was observed in patients with bacterial infection (21.4% severe itching in the bacterial group vs. 9.3% severe itching in the nonbacterial group, *P* < 0.0001). Significantly higher frequency of lesion distribution in the cubital fossa, popliteal fossa, side neck, ear, nape, shoulder and back, axillary, chest, abdomen, waist, upper limb, thigh, shank, foot, buttock, pudendum, and crissum was observed in patients with bacterial infection than those without bacterial infection (*P* < 0.04). On the contrary, significantly lower frequency of lesion distribution in the head and face was observed in patients with bacterial infection than those without bacterial infection (*P* < 0.02). No significant difference in lesion distribution in the eyelid and hand was found between the two groups (*P* > 0.42). Patients with bacterial infection also reported higher frequency of history of allergic diseases (i.e., asthma, allergic rhinitis, allergic conjunctivitis, and atopic dermatitis), dry skin, infantile eczema, and flexion dermatitis (*P* < 0.003; Table 1).

### 3.3. Multivariate Analysis of Risk Factors Associated with Clinically Suspected Bacterial Infection in Patients with Dermatitis and Eczema

As shown in Table 2, compared to atopic dermatitis, widespread eczema (OR = 1.50, 95% CI: 1.14-1.98; *P* < 0.001) and other eczema (OR = 1.42, 95% CI: 1.17-1.72; *P* < 0.001) were more likely to have bacterial infection. The itching grade was positively associated with bacterial infection (OR = 2.11, 7.04, and 12.3 in patients with mild, moderate, and severe itching, respectively; *P* < 0.001). Lesion distribution in the cubital fossa, popliteal fossa, ear, shoulder and back, axillary, foot, and pudendum was positively associated with bacterial infection (OR > 1.0, *P* < 0.05). History of asthma, allergic rhinitis, allergic conjunctivitis, atopic dermatitis, infantile eczema, and flexion dermatitis was positively associated with bacterial infection (OR > 1.0, *P* < 0.005), while a history of dry skin was reversely associated with bacterial infection (OR = 0.76, 95% CI: 0.64-0.90; *P* = 0.002).

## 4. Discussion

In the present study, we found that clinically suspected bacterial infection was very common in Chinese patients with dermatitis and eczema (47.0% of the patients). Previous studies showed that *S*. *aureus* was detected in 90% of chronic dermatitis lesions, which was in contrast to 5% *S*. *aureus* colonization in the skin of nonatopic individuals [19]. Our previous study revealed that secondary bacterial infection was more common in patients with adult AD than those with pediatric/adolescent AD (24.3% vs. 14.9%; *P* < 0.001) [5], which is consistent with the results of this study that patients with bacterial infection had significantly higher age of onset for dermatitis and eczema than those who had no bacterial infection (Table 1).

We found that widespread eczema and other eczema were easier to be infected with bacterial infection than atopic dermatitis (Table 2). Colonization of the skin with *Staphyloccoccus aureus* is well recognized in AD; we however found that widespread eczema was more susceptible than AD for infection. Higher rate of skin infection was reported in China than in other countries; this may be due to the clinical practice in China, in which the infection was diagnosed from clinical presentation rather than the use of bacterial culture, even in top-tier hospitals. The abuse of antibiotics for external use is thus a serious problem in China, especially in rural areas. Continuous efforts have been made to promote the use of bacterial culture for the diagnosis of skin infection. Relevant policies and regulations are also needed to prevent overuse of antibiotics.

Compared to atopic dermatitis, patients with widespread eczema had 50% increased risk to develop secondary bacterial infection, while patients with other eczema had 42% increased risk to develop bacterial infection. Notably, patients with bacterial infection had a significantly lower frequency of other eczema than those without bacterial infection before adjusting for other risk factors such as age of onset (Table 1). This highlights the advantages of multivariate analysis in which potential confounding factors are adjusted. Intriguingly, Wang et al. suggested that, in adults, AD is especially prone to suspected secondary bacterial infection compared to other types of dermatitis [5]. These conflicting results may be due to the differences in time of the survey. Our survey was conducted in the spring (i.e., March-May 2014) while the survey in the paper by Wang et al. was performed in the summer (i.e., July-September 2014).

Malfunction of the skin's normal barrier may contribute to the pathogenesis of AD [20]. Disruption of skin-barrier homeostasis may be due to lacking normal microbial skin diversity combined with a superabundance of staphylococcal species in patients with AD [21]. In the present study, the itching grade was found to be associated with bacterial infection (Table 2). There was a significant association between the itching grade and the risk for bacterial infection, possibly due to the more frequent scratching that leads to the severe damage to the skin [5]. This may increase the risk for bacterial infection. In this study, lesion distribution in the cubital fossa, popliteal fossa, ear, shoulder and back, axillary, foot, and pudendum was positively associated with bacterial infection, while the face and abdomen were reversely associated with bacterial infection (Table 2). Lesion distribution is probably related to the susceptibility to destruction of the skin's normal barrier function, leading to different risks for bacterial infection in patients with dermatitis and eczema. Our study also showed that history of asthma, allergic rhinitis, allergic conjunctivitis, atopic dermatitis, infantile eczema, and flexion dermatitis was positively associated with bacterial infection, while history of dry skin was reversely associated with bacterial infection (Table 2). Different medical histories might cause different extents of malfunction of the skin's normal barrier that is related to different risks for bacterial infection in dermatitis and eczema. Further investigations into these possible pathogeneses are needed.

The present study has a large sample size, and this is the first nationwide survey of secondary bacterial infections in Chinese patients with dermatitis and eczema. Compared to previous studies [5, 22], this study focused on the prevalence of bacterial infection and its association with various risk factors. Our results highlighted that the type of skin diseases (atopic dermatitis, widespread eczema, and other eczema), age of onset, course of disease, itching grade, lesion distribution, and history of dermatitis or eczema were risk factors for bacterial infection in dermatitis and eczema. Several limitations should be noted in this study: (1) study participants were recruited from 39 tertiary hospitals of 15 provinces and municipalities in China, and most of them are located in relatively developed regions in China. Most patients visiting these hospitals were in a better financial status and medical care than the average. As a result, the findings from this study might not represent the patients in developing regions in China. (2) As a hospital-based survey, selective bias is inevitable due to a nonhomogeneous population and differential spatial distribution. (3) Despite the criteria used for clinical diagnosis of secondary bacterial infection in this study are quite reliable [16], bacterial culture in laboratory is required to confirm the infection.

## 5. Conclusions

In summary, the present study showed the high prevalence of clinically suspected bacterial infection in patients with dermatitis and eczema. Our findings suggested that widespread eczema and other dermatitis or eczema were more likely to have bacterial infection than atopic dermatitis. The high rate of infection may attribute to the lack of corresponding bacterial detection, suggesting the need of guideline development in China to prevent overuse of topical antibiotics. Other clinical characteristics including age of onset, course of disease, itching grade, lesion distribution, and history of dermatitis or eczema were risk factors for secondary bacterial infection in dermatitis and eczema. Frequent follow-up and treatment strategies may need to be adjusted for these patients.

## Figures and Tables

**Table 1:** Demographic, clinical characteristics, univariate analysis of bacterial infection, and associated risk factors of 6208 patients with dermatitis and eczema.

Characteristic	Bacterial infection (*n* = 2918)	No bacterial infection (*n* = 3290)	*P* value
Gender, *n* (%)			0.14
Male	1525 (52.3)	1658 (50.4)	
Female	1393 (47.7)	1632 (49.6)	
Age, years (mean ± SD)	38.7 ± 17.5	36.9 ± 18.8	0.001
Age of onset, years (mean ± SD)	35.6 ± 17.2	34.1 ± 18.4	0.003
Course of disease, years (mean ± SD)	3.2 ± 4.5	2.9 ± 5.2	<0.0001
Diagnosis, *n* (%)			
Atopic dermatitis	507 (17.4)	468 (14.2)	0.0007
Widespread eczema	291 (10.0)	205 (6.2)	<0.0001
Other eczema	2120 (72.6)	2617 (79.6)	<0.0001
Itching, *n* (%)			<0.0001
None	14 (0.5)	116 (3.5)	
Mild	244 (8.4)	911 (27.7)	
Moderate	2035 (69.7)	1957 (59.5)	
Severe	625 (21.4)	306 (9.3)	
Lesion distribution, *n* (%)			
Cubital fossa	380 (13.0)	281 (8.5)	<0.0001
Popliteal fossa	397 (13.6)	311 (9.5)	<0.0001
Side neck	452 (15.5)	395 (12.0)	<0.0001
Head	442 (15.1)	569 (17.3)	0.02
Face	571 (19.6)	791 (24.0)	<0.0001
Eyelid	138 (4.7)	148 (4.5)	0.67
Ear	226 (7.7)	154 (4.7)	<0.0001
Nape	396 (13.4)	312 (9.5)	<0.0001
Shoulder and back	556 (19.1)	292 (8.9)	<0.0001
Axillary	283 (9.7)	125 (3.8)	<0.0001
Chest	402 (13.8)	296 (9.0)	<0.0001
Abdomen	441 (15.1)	402 (12.2)	0.001
Waist	522 (17.9)	423 (12.9)	<0.0001
Hand	755 (25.9)	881 (26.8)	0.42
Upper limb	803 (27.5)	757 (23.0)	<0.0001
Thigh	859 (29.4)	694 (21.1)	<0.0001
Shank	939 (32.2)	824 (25.0)	<0.0001
Foot	430 (14.7)	273 (8.3)	<0.0001
Buttock	262 (9.0)	205 (6.2)	<0.0001
Pudendum	156 (5.3)	123 (3.7)	0.002
Crissum	97 (3.3)	80 (2.4)	0.04
Medical history, *n* (%)			
Asthma	132 (4.5)	73 (2.2)	<0.0001
Allergic rhinitis	423 (14.5)	243 (7.4)	<0.0001
Allergic conjunctivitis	40 (1.4)	21 (0.6)	0.003
Atopic dermatitis	302 (10.3)	71 (2.2)	<0.0001
Dry skin	733 (25.1)	523 (15.9)	<0.0001
Infantile eczema	484 (16.6)	174 (5.3)	<0.0001
Flexion dermatitis	437 (15.0)	170 (5.2)	<0.0001

SD: standard deviation.

**Table 2:** Multivariate analysis of bacterial infection-associated risk factors in patients with dermatitis and eczema.

Risk factor	OR	95% CI	*P* value
Age of onset, years (mean ± SD)	1.01	1.00-1.01	0.001
Course of disease, years (mean ± SD)	0.99	0.98-1.00	0.026
Diagnosis, *n* (%)			
Atopic dermatitis	1.00 (ref.)		
Widespread eczema	1.50	1.14-1.98	<0.001
Other eczema	1.42	1.17-1.72	<0.001
Itching, *n* (%)			
None	1.00 (ref.)		
Mild	2.11	1.17-3.81	<0.001
Moderate	7.04	3.96-12.51	<0.001
Severe	12.3	6.83-22.28	<0.001
Lesion distribution, *n* (%)			
None	1.00 (ref.)		
Cubital fossa	1.29	1.03-1.62	0.024
Popliteal fossa	1.25	1.01-1.55	0.043
Side neck	1.17	0.99-1.39	0.060
Face	0.82	0.71-0.94	0.005
Ear	1.43	1.13-1.82	0.004
Shoulder and back	1.75	1.47-2.08	<0.001
Axillary	1.81	1.43-2.30	<0.001
Abdomen	0.78	0.66-0.93	0.004
Foot	1.50	1.25-1.79	<0.001
Pudendum	1.39	1.07-1.81	0.015
Medical history, *n* (%)			
None	1.00 (ref.)		
Asthma	1.88	1.35-2.61	<0.001
Allergic rhinitis	2.01	1.66-2.44	<0.001
Allergic conjunctivitis	2.52	1.40-4.51	0.002
Atopic dermatitis	4.17	3.05-5.70	<0.001
Dry skin	0.76	0.64-0.90	0.002
Infantile eczema	2.29	1.82-2.89	<0.001
Flexion dermatitis	1.43	1.12-1.83	0.005

CI: confidence interval; OR: odds ratio; SD: standard deviation.

## Data Availability

The datasets generated and analyzed during the present study are available from the corresponding author on reasonable request.
